# New insights into the pathophysiological mechanisms underlying cardiorenal syndrome

**DOI:** 10.18632/aging.103354

**Published:** 2020-06-19

**Authors:** Jin Wang, Weiguang Zhang, Lingling Wu, Yan Mei, Shaoyuan Cui, Zhe Feng, Xiangmei Chen

**Affiliations:** 1 Department of Nephrology, Chinese PLA General Hospital, Chinese PLA Institute of Nephrology, State Key Laboratory of Kidney Diseases, National Clinical Research Center for Kidney Diseases, Beijing Key Laboratory of Kidney Diseases, Beijing 100853, China

**Keywords:** cardiorenal syndrome, AKI, cardiac dysfunction

## Abstract

Communication between the heart and kidney occurs through various bidirectional pathways. The heart maintains continuous blood flow through the kidney while the kidney regulates blood volume thereby allowing the heart to pump effectively. Cardiorenal syndrome (CRS) is a pathologic condition in which acute or chronic dysfunction of the heart or kidney induces acute or chronic dysfunction of the other organ. CRS type 3 (CRS-3) is defined as acute kidney injury (AKI)-mediated cardiac dysfunction. AKI is common among critically ill patients and correlates with increased mortality and morbidity. Acute cardiac dysfunction has been observed in over 50% of patients with severe AKI and results in poorer clinical outcomes than heart or renal dysfunction alone. In this review, we describe the pathophysiological mechanisms responsible for AKI-induced cardiac dysfunction. Additionally, we discuss current approaches in the management of patients with CRS-3 and the development of targeted therapeutics. Finally, we summarize current challenges in diagnosing mild cardiac dysfunction following AKI and in understanding CRS-3 etiology.

## INTRODUCTION

Communication between the heart and kidney plays an important role in regulating fluid balance, metabolite excretion, and neuroendocrine function to maintain homeostasis [1]. The heart pumps fresh blood to maintain organ perfusion while the kidney filters the blood and concentrates metabolic waste, which is important for regulating blood volume and maintaining cardiac output. Cardiorenal syndrome (CRS) is defined as simultaneous bidirectional dysfunction of both the heart and kidney. The term ‘cardiorenal’ was first used by Thomas Lewis in a 1913 lecture entitled “Paroxysmal Dyspnoea in Cardiorenal Patients” in which he described his observation that patients with advanced kidney disease frequently developed dyspnea [1]. More recent studies have provided additional insight into the relationship between cardiac dysfunction and renal failure [2–4]. However, the etiology of CRS has not been fully elucidated [5]. Bongartz el al. expanded the definition of CRS to account for the role of renal dysfunction in heart failure in addition to the causal role of cardiac dysfunction in kidney disease [6]. The Acute Dialysis Quality Initiative (ADQI) currently defines CRS as a pathophysiological disorder in which acute or chronic dysfunction of one organ may induce acute or chronic dysfunction in the other organ [7].

Ronco et al. proposed a classification system that divides CRS into five subtypes based on acuity of onset and the primary organ involved (Table 1) [8]. CRS types 1 and 2 (CRS-1 and CRS-2) correspond to acute and chronic cardiorenal syndrome, respectively, whereas CRS types 3 and 4 (CRS-3 and CRS-4) correspond to acute and chronic renocardiac syndrome, respectively. CRS type 5 (CRS-5) is a secondary disease process that occurs in the context of other conditions such as diabetes, sepsis, and drug toxicity. In contrast, Hatamizadeh et al. proposed classifying CRS into seven distinct categories based on pathophysiologic mechanisms and the response to treatment strategies: 1) haemodynamic, 2) uraemic, 3) vascular, 4) neurohumoral, 5) anaemia and/or iron metabolism, 6) mineral metabolism, and 7) malnutrition-inflammation-cachexia (Table 2) [9].

**Table 1:** Cardiorenal syndrome classification system by Ronco et al.

**CRS General Definition:**
A complex pathophysiologic disorder of the heart and kidneys whereby acute or chronic dysfunction in 1 organ may induce acute or chronic dysfunction in the other organ.
**Types of CRS**
**CRS Type 1 (acute CRS)**
***Description*:** Abrupt worsening of cardiac function leading to AKI
***Examples*:** Acute coronary syndrome, acute decompensated heart failure
**CRS Type 2** (chronic CRS)
***Description*:** Chronic abnormalities in cardiac function causing progressive and permanent CKD
***Examples*:** Chronic heart failure, ischemic heart disease, hypertension
**CRS Type 3** (acute renocardiac syndrome)
***Description*:** Abrupt worsening of kidney function causing acute cardiac disorder
***Examples*:** Postsurgery AKI, acute glomerulonephritis, rhabdomyolysis
**CRS Type 4** (chronic renocardiac syndrome)
***Description*:** CKD contributing to decreased cardiac function, cardiac hypertrophy, fibrosis, and/or increased risk for adverse cardiovascular events
***Examples*:** Cardiac hypertrophy/fibrosis in CKD
**CRS Type 5** (secondary CRS)
***Description*:** Systemic condition causing both acute cardiac and kidney injury and dysfunction
***Examples*:** sepsis, diabetes mellitus

Abbreviations: AKI: acute kidney injury; CKD: chronic kidney disease; CRS: cardiorenal syndrome.

**Table 2:** Cardiorenal syndrome classification system by Hatamizadeh et al.

**CRS category (subclassified)**	**Manifestation**
**Haemodynamic** (acute/chronic)	Renal dysfunction due to cardiac output
**Uremic** (acute/chronic)	Uremic cardiomyopathy, Uremic pleuritis, Uremic pericarditis
**Vascular** (acute/chronic)	Coronary artery disease, Renal artery thrombosis, Renal artery stenosis
**Neurohumoral** (acute/chronic)	Abnormal serum calcium, potassium, magnesium and activated RAAS
**Anemia and/or iron metabolism** (acute/chronic)	Iron deficiency, Renal tubularinjury, Infection, Folate deficiency
**Mineral metabolism** (mostly chronic)	Vitamin D, Elevated FGF 23, Hypercalcemia, Hyperphosphatemia
**Malnutrition/inflammation-cachexia** (mostly chronic)	Cachexia, malnutrition and inflammation

Abbreviations: RAAS: Renin-Angiotensin system, FGF-23: Fibroblast Growth Factor 23.

In this review, we describe recent insights into the pathophysiological mechanisms underlying acute kidney injury (AKI)-induced cardiac dysfunction (CRS-3). Additionally, we discuss current approaches in the management of patients with CRS-3 including the development of targeted therapeutics.

### Epidemiology of AKI and CRS-3

AKI refers to acute kidney damage or failure. Although there is a consensus on the definition and diagnostic criteria for AKI, different terms are sometimes used to describe the pathology of the kidney injury. The primary criteria used to evaluate AKI stage are an increase in serum creatinine and a decrease in urine output (Table 3) [10]. Male sex, age, diabetes, blood pressure, a history of surgery, and atrial fibrillation are independent risk factors for AKI [11, 12].

**Table 3:** KDIGO classification criteria for acute kidney injury.

**Stage**	**Serum creatinine (Scr)**	**Urine output (UO)**
1	Baseline increase of 1.5 to 2 times in 7 days	<0.5 mL/kg/hour for 6–12 hours
2	Baseline increase of 2 to 3 times	<0.5 mL/kg/hour for ≥12 hours
3	≥4 mg/dL or a baseline increase >3 times or initiation of renal replacement therapy	<0.3 mL/kg/hour for ≥24 hours or anuria for ≥12 hours

Abbreviation: KDIGO: Kidney Disease Improving Global Outcomes.

The prevalence of AKI is increasing among hospitalized patients (4.9–7.2%) [13]. Severe AKI has been observed in over 40% of patients in the intensive care unit (ICU) [13]. The overall mortality for AKI patients is approximately 50%, but can be as high as 80% among ICU patients [14, 15]. AKI primarily results in acute tubular necrosis, a reduction in the glomerular filtration rate (GFR), and a decrease in renal perfusion. A previous multi-center study of AKI patients demonstrated that more than 60% of ICU patients also developed acute cardiovascular failure [16]. More recently, a multi-national, multi-center study of ICU patients with AKI indicated that the overall hospital mortality rate was 60.3% (95% confidence interval [CI]: 58.0–62.6%) and that cardiovascular-related death was the second leading cause of death [17]. Cardiogenic shock was shown to be an independent risk factor for hospital mortality (odds ratio [OR]: 1.41; 95% confidence interval [95% CI]: 1.05–1.90) [17].

The long-term effects of AKI on the cardiovascular system have also been investigated. A 5-year population-based study demonstrated that early- and late-onset post-operative AKI were independent risk factors for a composite cardiovascular endpoint that included myocardial infarction, heart failure, and other cardiovascular causes of mortality (hazard ratio [HR]: 1.41; 95% CI: 1.11–1.80) [18]. This finding was supported by a more recent study that explored the long-term risk of coronary events following AKI among 9,738 hospitalized patients who recovered from de novo dialysis-requiring AKI between 1999–2008 [19]. The incidence of coronary events was 19.8 per 1,000 person-years among AKI patients. They also found that AKI patients had an increased risk of coronary events (HR: 1.67; 95% CI: 1.36–2.04) and all-cause mortality (HR: 1.67; 95% CI: 1.57–1.79) after adjusting for chronic kidney disease and end-stage renal disease. Another study demonstrated that a single AKI event was associated with a two-fold and eight-fold increase in hospital mortality risk among patients with left or right ventricular dysfunction, respectively [20]. Several studies have indicated that AKI is an independent risk factor for 30-day readmission among heart failure patients (OR: 1.81; 95% CI: 1.35–2.39) [21]. ‘Frequent admitters’ were found to have longer lengths of stay (4.3 days vs. 4.0 days) and higher associated costs ($7,015 vs. $2,967) compared to non-frequent admitters among patients with repeat heart failure admissions [22].

The epidemiology of CRS-3 is not well understood. The incidence is likely underestimated as a consequence of a lack of early diagnostic criteria. Cardiac failure is typically diagnosed by echocardiography, but impaired cardiac function is frequently not observed until an advanced stage. However, early recognition of cardiac dysfunction is important given that AKI-mediated cardiac damage is frequently characterized by reduced diastolic function. The temporal relationship between AKI and cardiac damage has also not been fully elucidated. Patients with compensatory heart failure are likely to develop prerenal (functional) AKI as a consequence of reduced renal blood perfusion. If prerenal AKI persists for > 2 days, renal tubular cells will undergo cell death due to reduced perfusion. This can induce renal (structural) AKI, which can lead to decompensated heart failure. Thus, it can be difficult to determine the primary cause of CRS-3, particularly among elderly patients.

### Pathophysiology of CRS-3

### Hemodynamics

**Figure 1:**
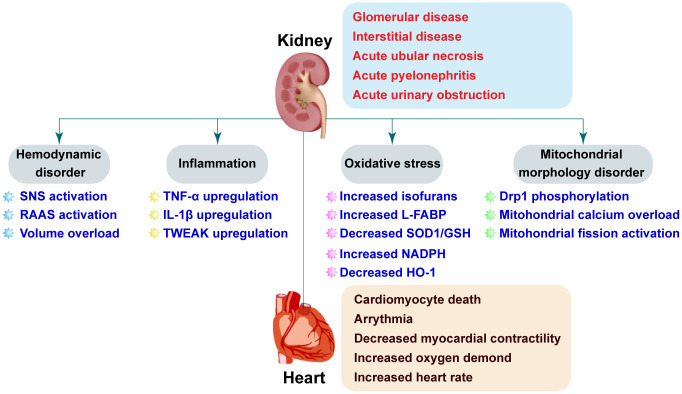
**Pathophysiologic mechanisms underlying CRS-3.** AKI is the initial insult in CRS-3 and has multiple potential etiologies. AKI may cause acute cardiac injury including heart failure, ischemia, and arrhythmia through both direct (e.g. SNS activation and RAAS) and indirect (e.g. volume overload, inflammation, oxidative stress, and mitochondrial dysfunction) effects.

AKI can cause cardiovascular damage through direct and1/or indirect mechanisms. Both hemodynamic and non-hemodynamic mechanisms have been proposed to explain crosstalk between the heart and kidneys (Figure 1). Hemodynamic disorders are characterized by activation of the sympathetic nervous system (SNS) and the renin-angiotensin-aldosterone system (RAAS) [23, 24]. SNS activation has been observed in AKI patients [25]. One study found that renal sympathetic nerve activity was elevated during renal ischemia and further increased during reperfusion [26]. Renal venous plasma norepinephrine concentrations were primarily elevated following reperfusion [26]. These findings may be explained in that the renal ischemia time was relatively short (28–30 minutes), and renal damage predominantly occurs during reperfusion. Elevated norepinephrine concentrations may be a delayed response to increased SNS activity. Although activation of the SNS enhances cardiac output by increasing myocardial contractility, higher levels of norepinephrine can cause increased cardiomyocyte oxygen consumption, cardiomyocyte death, and dysregulation of intracellular calcium. Additionally, activation of the SNS is followed by vasoconstriction and a reduction in the blood supply to the renal tubules, which exacerbates necrosis. Feedback mechanisms involving SNS activation and AKI can promote cardiac failure. Renal denervation can reverse AKI-induced histological alterations in the kidney and is therefore a potential therapeutic approach for preserving cardiac function following AKI [26].

RAAS activity is also elevated following AKI. Activation of the SNS stimulates β1-adrenergic receptors in the juxtaglomerular apparatus of the kidney resulting in reduced renal blood flow and activation of RAAS [27]. Juxtaglomerular cells in the juxtaglomerular apparatus secrete renin into the circulation in response to the reduction in renal perfusion. Renin cleaves angiotensinogen to yield angiotensin I (Ang I), which is then converted into angiotensin II (Ang II) by angiotensin converting enzyme. Increased Ang II concentrations are associated with systemic vascular resistance and water-sodium retention. These alterations can significantly augment cardiac preload and afterload. Ang II also exerts direct effects on cardiomyocytes by inducing hypertrophy and apoptosis [28, 29]. Interestingly, treatment with a RAAS inhibitor has been shown to protect against AKI and cardiac failure [30, 31].

AKI is characterized by a reduction in urine output and water-sodium retention. Increased blood volume, particularly venous congestion, results in an increase in cardiac preload. Volume overload causes myocardial edema, resulting in decreased myocardial contractility and left ventricular compliance [23, 24]. Right ventricular collagenase activity also increases in response to chronic interstitial edema, which contributes to myocardial remodeling [24]. Volume overload has also been shown to induce ventricular arrhythmias [32]. Although activation of the SNS and RAAS may be an adaptive response to AKI, dysregulated neurohumoral mechanisms also play an important role in promoting myocardial depression following AKI. Inhibition of SNS/RAAS activation is a standard treatment for patients with AKI.

### Inflammation

Inflammation is thought to be the primary non-hemodynamic mechanism that contributes to CRS-3 (Figure 1). A previous study demonstrated that two cytokines (TNF-α and IL-1) were released from ischemic kidney tissue into the blood, which resulted in an increase in TNF-α levels in myocardial tissue in a bilateral renal ischemia-induced mouse model of AKI [33]. Excessive TNF-α accumulation contributed to cardiac damage, as evidenced by an increase in the left ventricular end-diastolic and end-systolic diameters, and fractional shortening measured by echocardiography. Interestingly, TNF-α inhibition attenuated AKI-induced cardiac damage. These results are supported by a recent study by Alarcon et al., who explored the molecular basis of AKI-induced cardiac arrhythmias [34]. They found that *Nlrp3^-/-^* and *Casp1^-/-^* mice had normal QJ intervals and fewer ventricular arrhythmias compared to wide-type mice following renal ischemia/reperfusion (I/R) injury [34]. AKI was shown to cause IL-1β overproduction. Treatment with an IL-1β antagonist rescued the duration and amplitude of the calcium transient thereby protecting against ventricular arrhythmias. However, Toldo et al. demonstrated that exogenously administered IL-1β was associated with depressed myocardial contractility [35]. AKI-mediated inflammation can induce apoptosis in cardiomyocytes and promote cardiac fibrosis [36]. Sanz et al. demonstrated that tumor necrosis factor-like weak inducer of apoptosis (TWEAK) levels were elevated during AKI and that pharmacologic inhibition of TWEAK inhibited cardiac remodeling [36]. Collectively, these data suggest that inflammation-associated cytokines may be involved in transmitting signals from damaged kidney tissue to the heart resulting in myocardial depression.

Given the critical role of TNF-α in triggering AKI-related cardiovascular events and regulating myocardial contractility, several large multi-center trials have been performed to evaluate whether the TNF-α inhibitor etanercept could prevent heart failure. However, the results have suggested that etanercept has limited clinical efficacy and potentially results in worse patient outcomes [37].

### Oxidative stress

Oxidative stress results from an imbalance between the production and removal of reactive oxygen species (ROS). Four types of ROS have been defined: superoxide anion (O_2_^·-^), hydroxyl radical (^·^OH), hydrogen peroxide (H_2_O_2_), and hypochlorous acid (HOCl). ROS are involved in the regulation of cardiomyocyte viability and function. At physiological concentrations, ROS act as second messengers that are required for intracellular signal transduction. However, high levels of ROS are toxic to cells because they can induce protein and lipid oxidation. ROS can directly damage DNA and cause cell death through oxidative stress-mediated apoptosis. Overproduction of ROS is caused by increased oxidative metabolism and decreased antioxidative capacity. ROS are derived from both endogenous and exogenous sources. Mitochondrial electron transport, xanthine oxidase (XO), and nicotinamide adenine dinucleotide phosphate (NADPH) are the primary generators of endogenous ROS [38, 39]. Radiation, xenobiotics, the inflammatory response, cigarettes, and alcohol are the primary exogenous agents that generate ROS.

Antioxidative capacity is regulated by a series of antioxidants including superoxide dismutase (SOD), glutathione peroxidase (GPx), glutathione (GSH) and oxidized glutathione (GSSG) [40]. Several studies have revealed that antioxidants and ROS-induced lipid peroxidation products can function as biomarkers of AKI. Ware et al. evaluated the levels of circulating lipid peroxidation products in ICU patients by mass spectroscopy [41]. They found that plasma levels of F2-isoprostanes and isofurans were highly correlated with renal damage, suggesting that lipid oxidation is predictive of acute kidney injury. Interestingly, both of F2-isoprostanes [42] and isofurans [43] have been monitored as markers of chronic and acute cardiac damage, respectively. Liver-type fatty acid-binding protein (L-FABP), a cytotoxic oxidation product secreted by proximal tubular epithelial cells, was identified as a predictive biomarker of AKI [44, 45]. Other biomarkers of kidney damage have also been shown to be predictive of acute and chronic cardiac damage (e.g. heart failure) [46, 47]. Cardiorenal “connectors” such as erythrocyte superoxide dismutase (SOD1), GSH, and NADPH have also been used to measure kidney and heart injury [48–53].

Parenica et al. performed a retrospective controlled study to investigate the role of AKI in ST-elevation myocardial infarction. They found that circulating biomarkers of the nitric oxide (NO) pathway were associated with AKI [54]. Additionally, they showed that increased NOx was correlated with 3-month cardiovascular mortality. Heme oxygenase 1 (HO-1) is the rate-limiting enzyme that catalyzes the oxidative degradation of cellular heme to liberate free iron, carbon monoxide (CO), and biliverdin in mammalian cells. HO-1 regulates oxidative stress, activates autophagy, suppresses inflammation, and promotes cell cycle progression [55]. HO-1 deficiency sensitizes kidneys to I/R injury whereas upregulation attenuates AKI. Loss of HO-1 results in an increase in IL-6, which induces post-transcriptional phosphorylation of STAT3 in the heart and kidney following injury [56]. Two other studies have demonstrated that HO-1 is overexpressed in the heart [57, 58]. Because oxidative stress-related molecules can induce damage in the heart and kidney, therapies that reduce oxidative stress could be effective in CRS-3 (Figure 1). The endothelial nitric oxide synthase (eNOS) system primarily regulates vascular tone, which is important for cardiac function. Interestingly, eNOS levels are downregulated in the kidney after AKI [59]. It is possible that signals transmitted from renal endothelial cells to cardiac endothelia cells may explain the connection between AKI and cardiac dysfunction.

### Mitochondrial dysfunction

Mitochondria regulate cellular energy production through the tricarboxylic acid (TCA) cycle in which oxygen and glucose and broken down to generate ATP and H_2_O. Byproducts of the TCA cycle including ROS and lactic acid are released from the mitochondria into the cytoplasm where they regulate cell proliferation, pH, metabolism, and cell death. Mitochondria also play important roles in activating apoptosis. Mitochondrial dysfunction results in reduced ATP production and a reduction in cellular energy metabolism [60]. Damaged mitochondria can release pro-apoptotic factors into the cytoplasm and trigger apoptosis [61]. Additionally, interactions between mitochondria and other organelles such as the endoplasmic reticulum can activate apoptosis [62, 63].

The heart and kidney have high mitochondrial content compared to other organs, which makes them highly sensitive to the effects of mitochondrial dysfunction. Mitochondria-related oxidative stress can contribute to cardiorenal damage. Interestingly, alterations in mitochondrial morphology may play an important role in CRS-3. I/R injury can promote mitochondrial fission, which results in the division of a single mitochondria into two smaller units. Under normal physiological conditions, approximately 5% of renal tubules contain mitochondrial debris. However, approximately 50% or more of the tubules can be filled with fragmented mitochondria after AKI [64–66]. Mitochondrial fission is considered an early event in acute cardiac I/R injury that can induce cardiomyocyte apoptosis. Sumida et al. found that AKI induced mitochondrial fragmentation in heart tissue by promoting phosphorylation of dynamin-related protein 1 (Drp1) [67]. Inhibition of mitochondrial fission through administration of the Drp1 inhibitor Mdivi-1 attenuated I/R-mediated kidney damage and improved cardiac performance following AKI [67] (Figure 1).

Both oxidative stress and inflammation in the myocardium can induce mitochondrial fission [67]. Excessive mitochondrial fission results in a reduction in the mitochondrial membrane potential and the release of pro-apoptotic factors into the cytoplasm [68]. Mitochondrial fission can therefore function similarly to intracellular second messengers that sense extracellular signals (e.g. oxidative stress, inflammation, and hemodynamic changes) and then transmit these signals by undergoing changes in morphology. Alterations in mitochondrial shape suppress oxidative phosphorylation and activate mitochondria-dependent apoptosis, resulting in myocardial damage. Wang et al. demonstrated that mitochondrial calcium overload is also required for AKI-mediated mitochondrial fission in cardiomyocytes [68]. Additionally, Wang et al. reported that AKI induces phosphorylation of 1,4,5-trisphosphate receptor (IP3R) and upregulation of mitochondrial calcium uniporter (MCU) in cardiomyocytes [66]. Phosphorylation of IP3R and upregulation of MCU contributed to mitochondrial overload in cardiomyocytes, resulting in phosphorylation of Drp1 and mitochondrial fission [66]. These results indicate AKI activates Drp1-related mitochondrial fission in cardiomyocytes.

## CONCLUSIONS

In this review, we have summarized recent insights into the pathophysiological mechanisms underlying CRS-3. We propose a three-step mechanism that could explain the pathophysiology of CRS-3. Following AKI, the damaged kidney tissue first releases pro-inflammatory factors and oxidative metabolites into the circulation. Alterations in the neuroendocrine system also result in the secretion of several hormones into the blood. Next, kidney-derived biomolecules directly interact with receptors or adaptors on the surfaces of cardiomyocytes. It is also possible that they exert indirect effects on cardiomyocytes through other mechanisms. Finally, mitochondria respond to the kidney-derived biomolecules by changing morphology, which results in a reduction in ATP production and activation of apoptosis in cardiomyocytes. Our hypothesis has several limitations. First, the receptors or adaptors expressed on the surfaces of cardiomyocytes have not been verified. Second, mitochondria are not the only determinants of pathological alterations in cells. Intracellular acidosis, disorders of calcium metabolism, and mechanical pressure resulting from fluid overload may also trigger cardiomyocyte damage. Therefore, further studies of the relationships between mitochondria and other intracellular stress responses are required in order to understand the sequence of events that lead to cardiac damage following AKI.
